# A Polytetrafluoroethylene (PTFE) and Nano-Al_2_O_3_ Based Composite Coating with a Bacteriostatic Effect against *E. coli* and Low Cytotoxicity

**DOI:** 10.3390/polym14214764

**Published:** 2022-11-07

**Authors:** Dmitriy E. Burmistrov, Dmitriy A. Serov, Aleksander V. Simakin, Ilya V. Baimler, Oleg V. Uvarov, Sergey V. Gudkov

**Affiliations:** Prokhorov General Physics Institute of the Russian Academy of Sciences, 38 Vavilova St., 119991 Moscow, Russia

**Keywords:** aluminum oxide, polytetrafluoroethylene (PTFE), nanoparticles, composite, biocompatibility, antibacterial coating, cytotoxicity, biocompatibility

## Abstract

The problem of bacterial contamination through surfaces is important for the food industry. In this regard, there is a growing interest in new coatings based on nanoparticles that can provide a long-term antibacterial effect. Aluminum oxide nanoparticles are a good candidate for such coatings due to their availability and good biocompatibility. In this study, a coating containing aluminum oxide nanoparticles was produced using polytetrafluoroethylene as a polymer matrix—a polymer that exhibits excellent mechanical and physicochemical properties and it is not toxic. The obtained coatings based on “liquid Teflon” containing various concentrations of nanoparticles (0.001–0.1 wt%) prevented the bacterial growth, and they did not exhibit a cytotoxicity on animal cells in vitro. Such coatings are designed not only to provide an antibacterial surface effect, but also to eliminate micro damages on surfaces that inevitably occur in the process of food production.

## 1. Introduction

The problem of bacterial contamination is one of the most important for both the medicine and food industry, since it is posing a threat to human health. One of the main pathways for the spread of bacterial infections are surfaces on which bacterial cell adhesion is possible [1]. The classic examples are cutting boards used in the meat processing industry [2,3]. Defects inevitably occur during operation, making these surfaces vulnerable for bacterial cell colonization. In this regard, there is a growing interest in the creation of coatings that, on the one hand, can provide a prolonged antibacterial effect, and on the other hand, eliminate the damage and make the coating layer with necessary mechanical properties.

Currently, several approaches are being considered for creating surfaces with antibacterial properties. Nanomaterials are proposed as a promising approach [4,5,6], including nanosized particles of metals and metal oxides, which have a bacteriostatic and bactericidal effect along with low resistance in bacteria [7,8,9]. In general, metal oxide nanoparticles are popular and cheap to produce materials applied in modern life due to their unique properties and their high ratio of surface area to volume, which makes them highly reactive. Currently, the antibacterial action is well-known against a wide range of microorganisms, including antibiotic-resistant bacterial strains, nanomaterials based on metals and metal oxides. Examples of such nanomaterials are nanoparticles based on: ZnO [10,11,12,13], Fe_2_O_3_/Fe_3_O_4_ [14,15], TiO_2_ [16,17], Ag [18,19] and Ag_2_O [20], MgO [21,22], Cu [23,24] and CuO [25,26], and few others. It is noteworthy that there are also reports on the antibacterial activity of aluminum oxide nanoparticles, despite the non-reactivity of this material and its absence in usual biochemical processes of aluminum [27]. It is important to note that this nanomaterial demonstrates weak cytotoxicity at moderate concentrations with respect to normal human and animal cells [28,29]. This property makes aluminum oxide nanoparticles attractive for biomedical applications, including wound decontamination and coatings creation for implants [30], as well as for the use in the food industry: for creation of packaging [31] and antibacterial coatings. However, the use of pure nanoparticles is difficult and does not provide a long-term antibacterial effect. In this regard, it is proposed to use nanoparticles in the form of composite materials, as part of polymer matrices with the necessary physical and chemical properties. Polytetrafluoroethylene (PTFE, fluoroplast) is one of these materials. PTFE was first synthesized by Dr. Roy J. Plunkett at DuPont^®^ and it was given the commercial name Teflon^®^. It is known that this material has excellent hydrophobic, friction-proof, chemostable and thermostable properties due to the carbon-fluorine chemical bond [32]. Another important feature of fluoropolymers is their bio inertness and excellent biocompatibility [33]. Together, all these properties make PTFE an excellent candidate for the use as a carrier for the composite coatings, which are in demand both in the food industry [34,35] and in biomedicine [36].

In this work, a coating preparation is demonstrated based on PTFE doped with aluminum oxide nanoparticles preliminarily synthesized with laser ablation. The resulting coatings were characterized using a range of methods such as: DLS, CPS, EDS, ATM, modulation-interference microscopy (MIM), and spectrometry. It was found that the coating promoted the reactive oxygen species (ROS) formation, promoted oxidative damage to biomolecules (DNA and proteins) in vitro, and prevented the growth of *Escherichia coli*. However, the coating has not significantly affected the growth and development of animal cells even at high concentrations of nanoparticles in the composition.

## 2. Materials and Methods

### 2.1. Synthesis and Characterization of Al_2_O_3_ Nanoparticles

**Figure 1:**
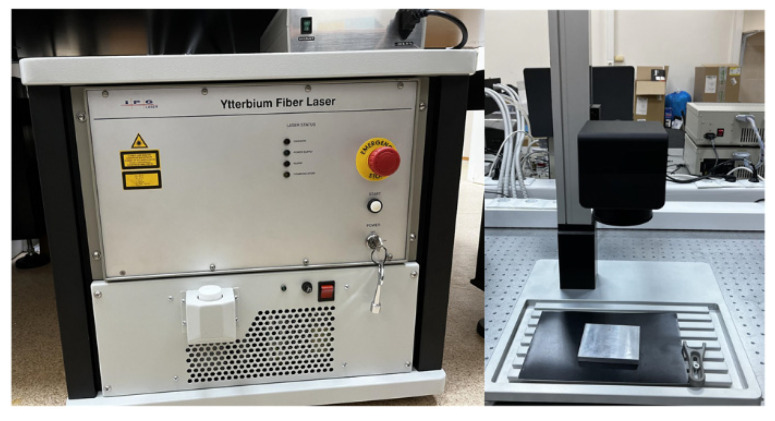
Installation for nanoparticles synthesis by laser ablation: laser-mechanical unit (**left**) and stand with galvano-mechanical scanner (**right**).

Aluminum oxide nanoparticles were synthesized using the method of laser ablation in the working fluid (deionized water). The target was a high purity aluminum plate A99 (EN AW-1199) containing at least 99.99% aluminum. The plate was immersed in a working liquid (ddH_2_O), whose layer above the metal target was about 1 mm. We used a P-Mark TT 100 (Pokkels, Moscow Russia) laser ablation installation based on a pulsed ytterbium fiber laser (Figure 1). The following laser parameters were used: wavelength, 1064 nm; pulse duration, 4–200 ns; pulse repetition rate, 20 kHz; average power, up to 20 W; pulse energy, 1 mJ. Irradiation time varied in the range from 5 to 20 min.

The characterization of the obtained nanoparticles was carried out using the Malvern Zetasizer Ultra (Malvern Panalytical Ltd., Malvern, UK) to determine the hydrodynamic diameter and zeta potential of the nanoparticles. The features of the parameters recording were described earlier. A disk analytical centrifuge CPS 24000 (CPS Instruments, Prairieville, LA, USA) was used to estimate the diameter of the nanoparticles [37]. The morphological features of nanoparticles (shape, topology), as well as the elemental composition of nanoparticles, were studied using a Libra 200 FE HR transmission electron microscope (Carl Zeiss, Jena, Germany) in combination with a JED-2300 energy dispersive X-ray spectrometer. To assess the morphological features of the surface of the obtained composite coatings, a Seiko SII NPX-200 atomic force microscope (Seiko Instruments Inc., Tokyo, Japan) was used [38]. The distribution of nanoparticles in PTFE was assessed using a MIM-321 interference modulation microscope (Amphora laboratories, Moscow, Russia). Additionally, to confirm the composition of the obtained nanoparticles, the spectrum of aqueous NPs colloids was recorded using Cintra 4040 (GBC Scientific Equipment, Braeside, Australia).

### 2.2. Hydrodynamic Diameter and ζ-Potential of Nanoparticles Measurements

Hydrodynamic size distributions and concentrations of nanoparticles were obtained by dynamic light scattering (DLS). Malvern Zetasizer Ultra (Malvern Panalytical Ltd., Malvern, UK) was used for measurements. Measurements were taken at 12 mm o.d. square polystyrene cuvettes. To obtain size distribution by intensity, the following constants were used: water viscosity, 0.8872 mPa·s; refractive index, 1.33, Al_2_O_3_ refractive index, 1.77; absorption, 0.001. Automatic sets from ZS Xplorer software were used during measurements. Ζ-potential measurements were obtained by Malvern Zetasizer Ultra (Malvern Pana-lytical Ltd., Malvern, UK), using Folded Capillary Zeta Cell, at the temperature 25 °C. All measurements were made using automatic attenuation and automatic measurement process (the range of runs for each measurement from 10 to 100) from ZS Xplorer. The pause between repeats was 60 s. The equilibration time was 120 s.

### 2.3. Composite Fabrication & Preparation of Coatings from Composite Material

After the synthesis of nanoparticles, the solvent was replaced by centrifugation. The colloidal solution of nanoparticles was centrifuged using Sigma 3–16KL (Sigma Laborzentrifugen GmbH, Osterode am Harz, Germany) centrifuge with a 12158 rotor for 40 min at 7017× *g*, then the supernatant was carefully replaced with acetone. The procedure was carried out at least 3 times. Next, the resulting colloidal solution was mixed with LG-32 LN fluoroplastic varnish (Plast Polymer-Prom, Saint-Petersburg, Russia) to a final concentration of nanoparticles of 0.1, 0.001, and 0.0001 wt%. Varnish LG-32 LN is a PTFE dissolved in a mixture of acetone, butyl acetate, cyclohexanone and toluene in a ratio of 25:40:10:25 mass parts. Drops of a solution of nanoparticles in a varnish with a volume of 500 μL were placed on round degreased 25 mm glasses. Before the start of the experiments, the coatings were dried for 48 h in a fume hood. For microbiological and cytotoxic studies, composite-coated slides were pre-disinfected by soaking in 70% ethanol for 2–3 h.

### 2.4. Hydrogen Peroxide Concentration Measurement

To estimate the concentration of the formed H_2_O_2_, the chemiluminescence method of the luminol-p-iodophenol-horseradish peroxidase system was used. Chemiluminescence was recorded using a highly sensitive chemiluminometer Biotox-7A-ultrasound (ANO “Engineering Center-Ecology”, Russia). The calibration and registration procedures were carried out according to the protocols described in more detail earlier [39,40]. Composite material samples containing various concentrations of Al_2_O_3_-NPs in the composition (0.001–0.1 wt%) in the form of films with a size of 10 × 10 mm and a thickness of 200 microns were placed in polypropylene vials (Beckman, CA, USA). After incubation in 20 mL of water, 1 mL of a pre-prepared “counting solution” was added to the sample. This solution contained 1 mm of tris-HCl buffer, pH 8.5, 50 microns of p-iodophenol, 50 microns of luminol, 10 nM of horseradish peroxidase. In the “Control” group, the experiment was conducted without a sample. The sensitivity of this method made it possible to determine H_2_O_2_ at a concentration of <1 nM [41].

### 2.5. Hydroxyl Radicals Concentration Measurement

The concentration of OH radicals formed in aqueous solutions was determined by reaction with coumarin-3-carboxylic acid (CCA), the product of which is hydroxycoumarin-3-carboxylic acid (7-OH-CCA); 7-OH-CCA is a fluorescent probe providing quantitative determination of OH radicals [42]. Briefly, 0.2 M PBS (pH 7.4) was added to the CCA solution in water (0.5 mM, pH 3.6). Further, coating samples containing various concentrations of Al_2_O_3_-NPs in the composition (0.001–0.1 wt%) were added to the vials in the form of films with a size of 10 × 10 mm and a thickness of 200 microns. In the “Control” group, the experiment was conducted without a sample. Further, the polypropylene vials with samples and reagents were heated in a thermostat at a temperature of 80.0 ± 0.1 °C for 2 h. Fluorescence of 7-OH-CCA was recorded using JASCO 8300 (JASCO, Japan) at λ_ex_ = 400 nm (excitation wavelength), λ_em_ = 450 nm (radiation wavelength). Commercial 7-OH-CCA (Sigma-Aldrich, St. Louis, MO, USA) was used for calibration [43].

### 2.6. Long-Lived Reactive Protein Species Concentration Measurement

The chemiluminescence method is effective and sensitive for the determination of free radical reactions. The interaction of radicals is accompanied with the release of energy in the form of emitted light quanta [44]. In this case, the interaction of radicals releases the energy emitted in the form of light quanta. Chemiluminometer Biotoks-7A (ANO “Engineering Center-Ecology”, Russia) was used to study long-lived reactive forms of proteins by measuring the chemiluminescence of protein solutions with increasing temperature. The measurements were carried out in the dark at room temperature in 20 mL plastic polypropylene vials (Beckman, Brea, CA, USA). All samples were stored in the dark at room temperature for 30 min after exposure. Unheated protein solutions were used as controls. A more detailed description of the method was presented in the published paper of Sharapov et al. [45].

### 2.7. Quantitative Determination of 8-Oxoguanine Using the ELISA Method

To quantify 8-oxoguanine in DNA, a non-competitive enzyme-linked immunosorbent assay (ELISA) was used using monoclonal antibodies specific for 8-oxoguanine (anti-8-OG antibodies). DNA samples (350 μg/mL) were denatured by boiling in a water bath for 5 min and cooled on ice for 3–4 min. Aliquots (42 μL) were applied to the bottom of the wells of the ELISA plates. The DNA was immobilized using a simple adsorption procedure with incubation for 3 h at 80 °C until the solution became completely dry. Non-specific adsorption sites were blocked with 300 μL of a solution containing 1% skimmed milk powder in 0.15 M Tris-HCl buffer, pH 8.7 and 0.15 M NaCl. Next, the plates were incubated at room temperature overnight (14–18 h). The formation of an antigen-antibody complex with antibodies against anti-8-OG (at a dilution of 1:2000) was carried out in a blocking solution (100 µL/well) by incubation for 3 h at 37 °C. Then, it was washed twice (300 µL/well) with 50 mM Tris-HCl buffer (pH 8.7) and 0.15 M NaCl with 0.1% Triton X-100 after 20 min incubation. Next, a complex with a conjugate (anti-mouse immunoglobulin labeled with horseradish peroxidase (1:1000) was formed by incubation for 1.5 h at 37 °C in a blocking solution (80 µL/well). Then the wells were washed 3 times as described above. Next, a chromogenic substrate containing 18.2 mM ABTS and hydrogen peroxide (2.6 mM) in 75 mM citrate buffer, pH 4.2 (100 µL/well) was added to each well [46]. Reactions were stopped by adding an equal volume of 1.5 mM NaN_3_ in 0.1 M citrate buffer (pH 4.3) upon reaching the color. The optical density of the samples was measured on a plate photometer Titertek Multiscan, (Titretek, Helsinki, Finland) at λ = 405 nm.

### 2.8. Antibacterial Activity Assay

The antibacterial activity of the obtained PTFE/Al_2_O_3_-NPs coatings was evaluated against gram-negative bacteria Escherichia coli. Sterile samples of coatings in the form of films with an area of 20 cm^2^, pre-soaked for several hours in 70% ethanol, were put on a sterile hoop, on which a liquid nutrient medium LB was then placed with a known initial number of colony-forming units (CFU) calculated using a counting chamber. The resulting known amount of CFU was used for further calibration. A wrap-sealed film sample with a cell suspension in LB broth was placed in an ES-20 shaker incubator (Biosan, Riga, Latvia) and cultured at 37 °C, ~150 rpm for 24 h. The optical density of the bacterial suspension was estimated using a UV5Nano Excellence drop spectrometer (Mettler Toledo, Columbus, OH, USA). The optical density measured at 600 nm reflected the concentration of cells in the nutrient medium per unit volume [47].

### 2.9. Protocol of Manipulations with Animals

All manipulations with animals and isolation of fibroblast cells were performed at the Institute of Cell Biophysics, Russian Academy of Sciences (Pushchino, Russia). Male mice of the BALB/c line weighing 22–30 g were used in the study. The animals were purchased from the Pushchino laboratory animal nursery (FIBKh RAS, Pushchino, Russia). All experiments with laboratory animals were carried out in accordance with the regulatory legal act of the health laboratory of the Russian Federation No. 199-n “General cases of applying the rules of good practice, international legal norms for use in an-own conditions” ETS No. 123 “On the protection of vertebrate animals and scientific research” and, according to indications, with laboratory animals of the IKB RAS No. 57.30.12.2011. Animals were adapted to normal conditions, received water and food without restrictions.

### 2.10. Preparation of Primary Cultures of Mouse Lung Fibroblasts

Isolation and preparation of cell culture of isolated fibroblasts from the lungs of mice was carried out according to the standard protocol. Euthanasia of a 2–3-month-old mouse was carried out by cervical dislocation. Then, using scissors and tweezers, the chest was dissected and the lungs were removed, which were placed in a Petri dish with a sterile PBS solution. The organs were chopped with sterile scissors into pieces with a volume of ~1 mm^3^. The shredded organs were incubated for 1 h in 25 mL of DMEM containing 0.2% (weight/volume) type II collagenase at 37 °C. Collagenase was then inhibited with 20% FBS. Tissue pieces incubated in collagenase solution were resuspended by pipetting and then passed through an EASTstrainer™ sieve with a mesh size of 70 μm (Greiner bio-one, Kremsmunster, Austria). Cells were washed with double centrifugation at 350× *g* for 5 min in DMEM. The isolated cells were further cultivated in TC T-25 flasks (TPP, Switzerland) in DMEM:F12 medium (PanEco, Moscow, Russia). The medium was supplemented with 10% FBS, 100 U/mL penicillin, 2 mM L-glutamine, 100 µg/mL streptomycin, also obtained from PanEco (PanEco, Russia). When ~90% confluency was reached, the cells were removed from the flask surface with 0.05% Trypsin EDTA solution (PanEco, Moscow, Russia), and 10% FBS was used to inactivate trypsin. Cells were washed with PBS and suspended in culture medium to obtain a final concentration of 300,000 cells/mL.

### 2.11. Cytotoxicity Assay

Samples of PTFE/Al_2_O_3_-NPs composite coatings were pre-sterilized in 70% ethanol. Coated Ø25 mm round slides Menzel Glaser (Thermo Fisher Scientific, MA, USA) were placed in the wells of a 6-well plate (TPP, Trasadingen, Switzerland). Cell suspension was applied to each coating sample (50 μL each). The cultures were incubated for 45 min in a CO_2_ incubator for better cell adhesion. Then, 1 mL of nutrient medium was carefully added to each well. Cell cultures were incubated in a CO_2_ incubator S-Bt Smart Biotherm (Biosan, Riga, Latvia) (37 °C, 5% CO_2_) for 72 h.

Cell viability was assessed using fluorescence microscopy. Mouse fibroblast cells were incubated for 72 h on the surface of a PTFE/Al_2_O_3_-NPs coating of a composite material containing 0.1 wt% Al_2_O_3_-NPs. The initial number of cells in the suspension placed on the surface of the material was ~50 thousand cells per sample. Hoechst 33342 and propidium iodide (PI) dyes were used to evaluate cytotoxicity. Fluorescence microscopy with Hoechst 33342 and PI staining is a widely used standard cell viability test (including NP cytotoxicity assays and drug screening) [48]. Immediately after incubation, the coating sample with cultured cells was placed in a coverslip chamber (RC-40LP, Warner Instruments, Holliston, MA, USA), washed thoroughly with PBS, and stained with 5 µg/mL Hoechst 33342 and for 30 min at 37 °C. Then the sample was washed with PBS and stained with 2 μM PI (ThermoFisher, Waltham, MA, USA) for 1 min. The samples were analyzed using a DMI4000 B fluorescence microscope (Leica Microsystems, Wetzlar, Germany) equipped with an SDU-285 digital camera (SpetsTeleTekhnika, Moscow, Russia). Fluorescence spectra were recorded at excitation/emission wavelengths: 350/470 for Hoechst 33342 filter cube D (Leica Microsystems, Wetzlar, Germany), 540/590 for PI (filter cube TRITC Leica, Germany). Light emitting diodes (LED) M375D2m, M490D3 (Thorlabs, Newton, NJ, USA) and white LED (Cree Inc., Durham, NC, USA) were used as light sources for excitation of Hoechst 33342 and PI fluorescence. All images were taken with the same LED current: 100 mA for M375D2m (Hoechst 33342), 250 mA for white LED (PI). The exposure time in all experiments was the same: 500 ms for Hoechst 33342 and 700 ms for PI. The detector gain was x423 and was the same for all fluorophores and experimental conditions.

Data collection and control of the microscope setup were performed using the WinFluorXE software (J. Dempster, Strathclyde Electrophysiology Software, University of Strathclyde, UK). The data was collected as 12-bit grayscale images. Subsequent analysis was performed using ImageJ2 (Fiji) software (NIH, Bethesda, MD, USA). For each variant of the experiment, at least five samples were analyzed. At least 200 cells were analyzed in each sample.

Regions of interest (ROI) were determined using standard ImageJ “Threshold” and “Particle Analysis” automated procedures. The parameters for analysis and determination of ROI were chosen as a result of preliminary experiments. For a 1392 × 1024-pixel image obtained with a value of ×20, the following parameters were used: “size” = 100–750 and “circularity” = 0.10–1.00. The nuclei had different Hoechst 33342 and PI fluorescence intensities. In order to ensure that all nuclei are included in the analysis, we ran a series of Threshold and Particle Analysis procedures on each image. The images were converted to 8-bit prior to determining the ROI. Threshold levels were from 5 to 255 a.u. with a step of 5 a.u., ROIs were saved as binary masks, then all ROIs were combined together with the removal of duplicates, all procedures were integrated into automated macros.

### 2.12. Statistic

Experimental data analysis and statistical processing were performed using the SigmaPlot, Origin, and GraphPad Prism software packages. Data are presented as mean values with standard errors of the mean. Data from at least three independent experiments were used for averaging.

## 3. Results and Discussion

**Figure 2:**
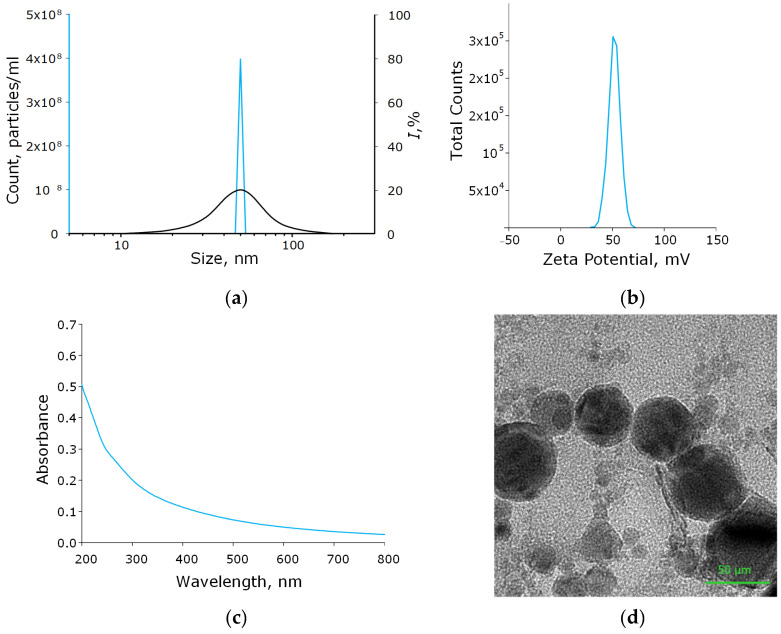
Physicochemical characteristics of the Al_2_O_3_-NPs colloidal solution. Concentration (solid blue line) and size distribution (black line) of Al_2_O_3_-NPs (**a**); ζ-potential of NPs (**b**); optical absorption spectrum of the obtained colloidal solution of NPs (**c**); TEM image of aluminum oxide nanoparticles (**d**).

**Figure 3:**
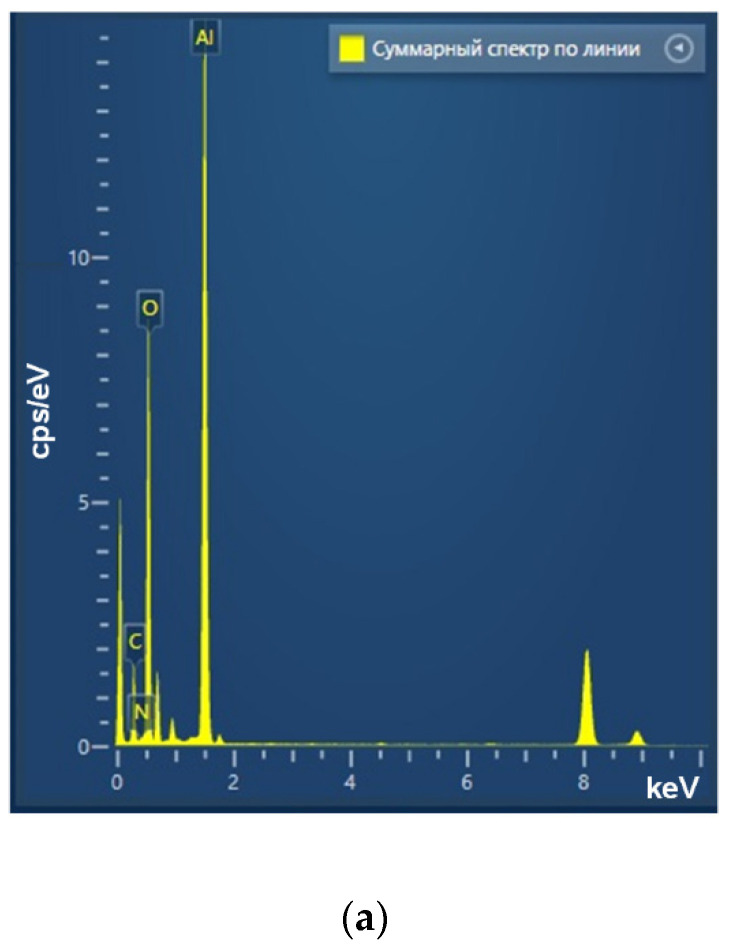

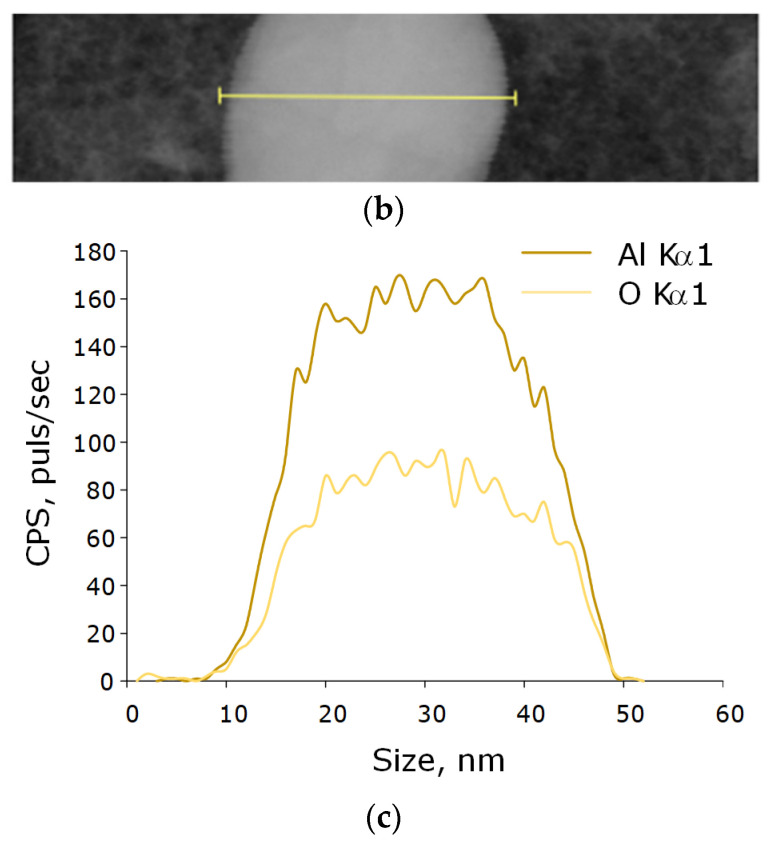
Elemental analysis of aluminum oxide NPs using EDX. EDX spectra (**a**) TEM image of single NP analysis with measurement site (**b**); profile of the selected nanoparticle of Al Kα1 and O Kα1 (**c**).

Using the laser ablation method, the aluminum oxide nanoparticles were obtained. These nanoparticles had an average size of about 50 nm, confirmed with DLS and CPS; ζ-potential of the order of 50 mV, which indicates the stability of the colloidal solution; the optical absorption spectrum of the nanoparticles made it possible to establish that the obtained colloidal solution contained Al_2_O_3_; using TEM, it was found that NPs had a spherical morphology (Figure 2a–d). As a result of energy dispersive X-ray spectroscopy (EDX) analysis, it was shown that the obtained aluminum oxide nanoparticles had the required chemical purity and did not contain impurities (Figure 3a–c).

**Figure 4:**
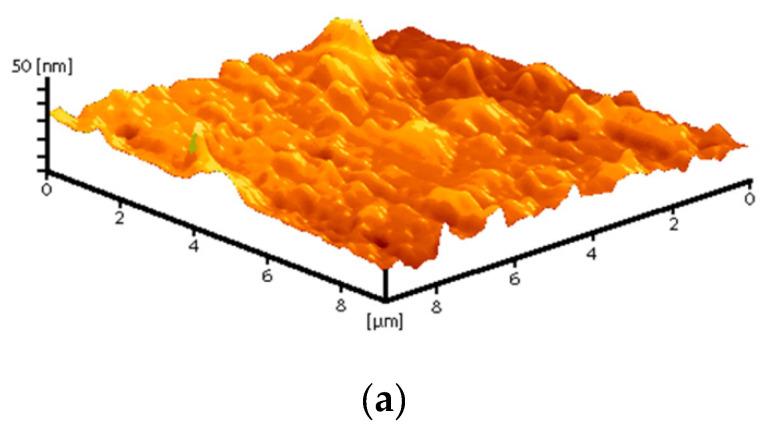

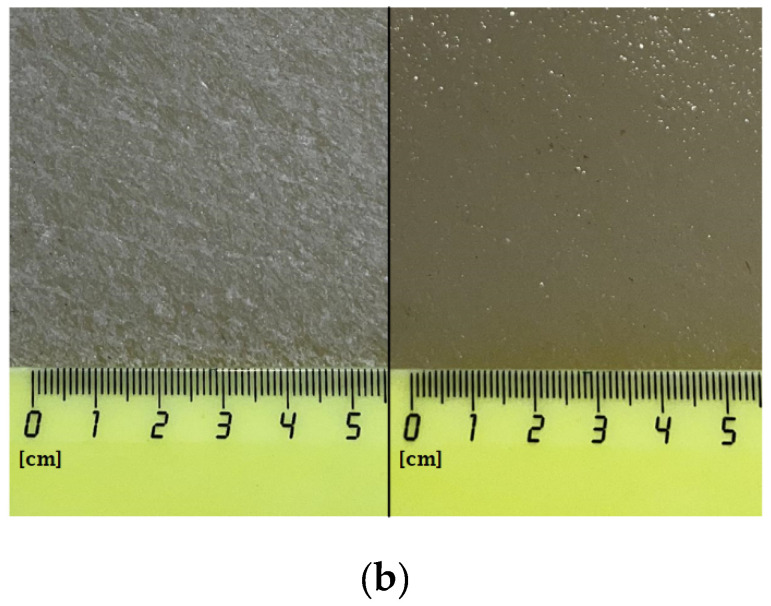
Atomic force microscopy image of the composite coating surface (**a**); photo of a section of a PTFE sample with a damaged surface (**b**): before applying the PTFE/Al_2_O_3_-NPs coating (**left**) and after (**right**).

Composite materials were prepared by mixing a colloidal solution of nanoparticles with a solution of PTFE in a mixture of solvents (PTFE varnish). Since the aqueous colloidal solution of nanoparticles is immiscible in nonpolar solvents that are part of the PTFE varnish, the replacement of the solvent was carried out through several centrifugations and replacement of the supernatant with acetone (see Section 2.3). The dried coatings were visually smooth and uniform. AFM also confirmed the absence of significant defects (grooves, cracks, protrusions) in the microstructure of the coating (Figure 4a). The composite coating was also applied on the surface of PTFE with surface damage. The material was found to have the ability to fill gaps and had good adhesion to the surface (Figure 4b).

**Figure 5:**
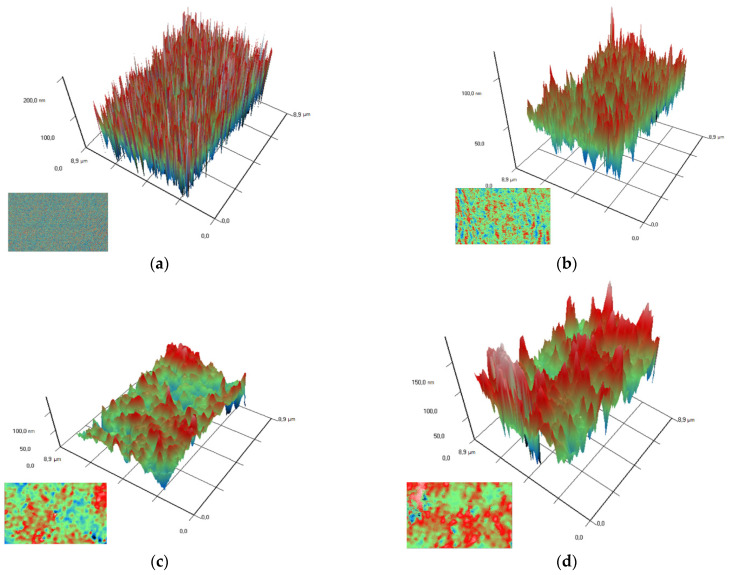
Images of the surfaces of polymer/Al_2_O_3_ -NPs composite coatings with different dopant content (PTFE without NPs (**a**); 0.001 wt% (**b**); 0.01 wt% (**c**); 0.1 wt% (**d**)) obtained using modulation interference microscopy. *X*- and *Y*-axis surface size (µm); *Z*-axis surface topography (nm). The image in the lower left corner for each figure is the image above, showing the distribution of the dopant in the matrix.

Using the MIM method (modulation-interference microscopy), it is possible to study not only the surface of a material, but also the distribution of clusters in optically inhomogeneous media. The refractive index of PTFE is 1.4 at a wavelength of 405 nm, while the refractive index of Al_2_O_3_-NPs is 1.77 at a wavelength of 405 nm, which made it possible to successfully detect the distribution of nanoparticles in the polymer composition. It was found that Al_2_O_3_-NPs are localized in the polymer in the form of clusters that increase in size with increasing concentration of nanoparticles (Figure 5).

**Figure 6:**
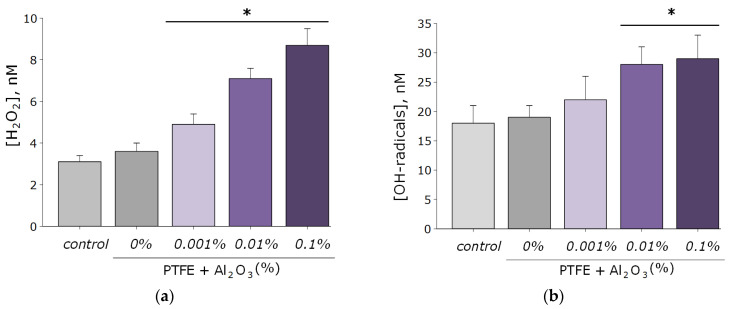
Results of evaluating the effect of PTFE/Al_2_O_3_-NPs on the formation of hydrogen peroxide (**a**) and hydroxyl radicals (**b**). *—statistically significant differences compared to the “control” group (without sample), (*p* < 0.05). Data are presented as the mean ± standard error of the mean.

As known, reactive oxygen species are highly reactive compounds that exhibit biological activity and damage macromolecules (membrane lipids, proteins, and nucleic acids) [49,50]. In connection with this, the ability of the resulting PTFE/Al_2_O_3_-NPs composite coatings to form ROS (H_2_O_2_ and OH-radicals). As a result, coatings increase the rate of formation of the considered ROS only at NPs concentrations of 0.01 and 0.1 wt% (Figure 6a,b). At the same time, the concentration of hydrogen peroxide in the group with the maximum content of Al_2_O_3_ NPs (0.1 wt%) was approximately three times higher than when using PTFE without the addition of Al_2_O_3_ NPs, and the concentration of hydroxyl radicals was 1.6 times higher. It is noteworthy that the degree of formation of Al_2_O_3_-NPs ROS is much lower than that of other nanoparticles characterized by higher cytotoxicity, such as ZnO [12,51,52].

**Figure 7:**
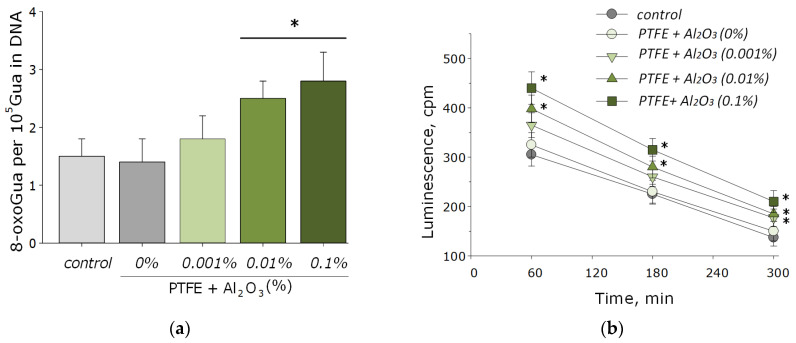
The effect of PTFE/Al_2_O_3_-NPs composites on the formation of 8-oxoguanine in DNA (**a**), as well as on the dynamics of the formation of reactive long-lived forms of proteins (**b**). *—statistically significant differences compared to the “control” group (without sample), (*p* < 0.05). Data are presented as the mean ± standard error of the mean.

Nucleic acids are often the main target for ROS, especially in bacterial cells [53]. The main product of oxidative DNA damage, 8-oxoguanine, often causes nucleotide mismatches with adenine [54]. It is well-known that the accumulation of 8-oxoguanine in DNA plays a role in the cancer development and in the process of aging [55]. On the other hand, it is known that ROS can cause oxidative damage to protein macromolecules with the formation of active long-lived forms of proteins that can cause cellular mutations and transformations, as well as being a source of secondary free radicals that cause subsequent damage to biomolecules [44,46]. It was found that PTFE coatings functionalized with the addition of Al_2_O_3_-NPs promoted the formation of 8-OG in DNA in vitro, as well as long-lived reactive forms of proteins. At an Al_2_O_3_-NPs concentration of 0.01 wt%, an increase of the rate of formation of 8-oxoguanine in DNA by 1.5-times was observed; at a concentration of 0.1 wt%, it was ~two-times (Figure 7a). The half-life of the active forms of proteins did not change after the addition of Al_2_O_3_-NPs and was about 4–5 h (Figure 7b). The effect was observed only for materials containing high concentrations of NPs (0.001 and 0.01 wt%).

**Figure 8:**
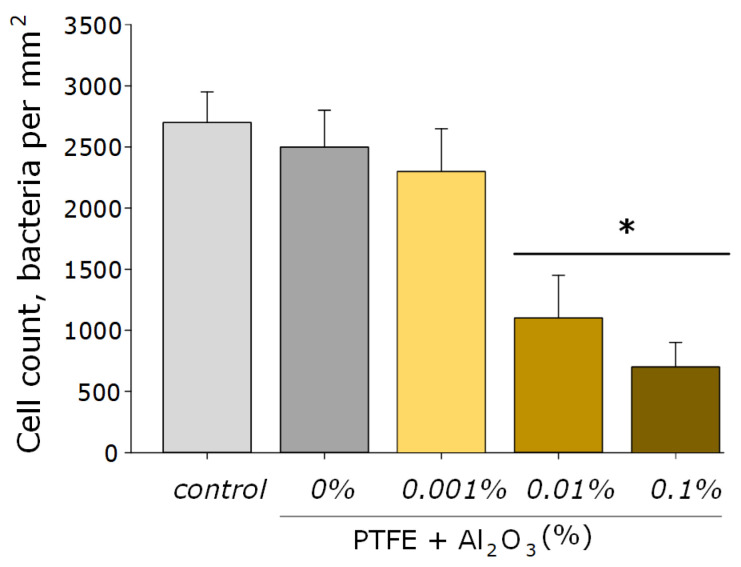
The microbiological study of the obtained PTFE/Al_2_O_3_-NPs composites containing various dopant concentrations in relation to *E. coli*. *—statistically significant differences compared to the “control” group (without sample), (*p* < 0.05). Data are presented as the mean ± standard error of the mean.

As for many nanoparticles of metals and metal oxides, Al_2_O_3_-NPs are characterized by antibacterial activity. This effect is provided by the mechanical action of these nanoparticles on the cell wall and the membrane of the bacterial wall, the formation of aluminum ions that bind to the membrane and intracellular proteins of the bacterial cell, as well as by the ROS-mediated pathway [27]. It was found that the obtained PTFE/Al_2_O_3_-NPs composite coatings containing 0.001 and 0.01 wt% NPs had a bacteriostatic effect against Gram-negative *E. coli* (Figure 8). The table below (see Table 1) presents several other studies, also illustrating the successful use of Al_2_O_3_-NPs as an antibacterial agent, as well as ways to improve their effectiveness.

**Figure 9:**
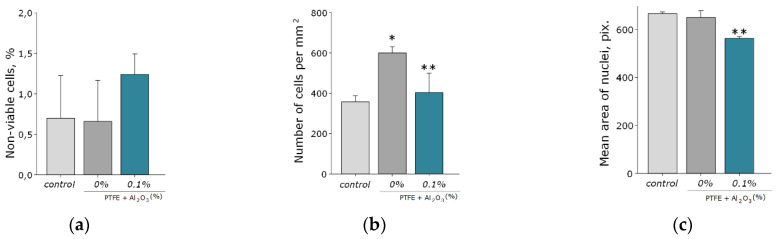

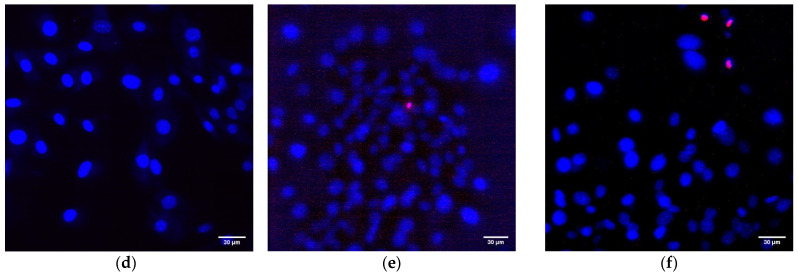
Evaluation of the cytotoxic effect of PTFE/Al_2_O_3_-NPs composite coating on the growth and development of mouse fibroblast cultures (DIV 4). Proportion of non-viable cells in culture (**a**); the number of cells per unit area (**b**), the average area of the cell nucleus (**c**). *—statistically significant differences compared to the “control” group (*p* < 0.05), **—statistically significant differences compared to the “PTFE + 0%Al_2_O_3_” group (*p* < 0.05). Data are presented as mean ± standard error of mean. Photomicrographs of cell cultures obtained after staining with Hoechst and PI (merges). Cells growing for 72 h on the surface: glasses (control) (**d**); PTFE without added NPs (0%) (**e**); PTFE/Al_2_O_3_-NPs composite coating containing 0.1 wt% NPs (**f**). Nuclei of all cells are stained blue, dead cells are stained pink.

Another important property of Al_2_O_3_-NPs is low cytotoxicity compared to other nanomaterials based on metals and metal oxides, which is confirmed in a number of experimental research studies [28,66,67]. In this study, the analysis of cytotoxicity of the obtained PTFE/Al_2_O_3_-NPs with a high content of Al_2_O_3_-NPs (0.1 wt%) showed that the coating did not affect the growth and development of mouse fibroblast cells in vitro; it was revealed in the absence of differences in the count of non-viable cells, as well as the density of cultures, which were kept at the level of “control” values (Figure 9a,b). The percentage of PI-positive cells in the “PTFE/Al_2_O_3_-NPs 0.1%” group was 1.23 ± 0.25% (Figure 9a). The density of cells growing on the composite coating also did not differ from the control group and it was 404 ± 97 cells/mm^2^. The cells growing on the composite coating did not form a solid monolayer, they were located diffusely and formed local clusters. It was also found that on the surface of PTFE without nanoparticles, the number of cells was higher compared to the PTFE/Al_2_O_3_-NPs composite material (Figure 9b). This effect is possible due to the good adhesive characteristics of PTFE for a number of cells [68,69] and also due to uneven distribution of cells on the surface. It is known that PTFE is a non-toxic material used for medical devices covering [70]. Additionally, it has been found a slight decrease in the average area of the nuclei of cells growing on the surface of the PTFE/Al_2_O_3_-NPs coating (Figure 9c). Currently, the exact mechanisms of changes in the size and shape of the nuclei, as well as the physiological consequences of these processes, are poorly understood [71]. On the one hand, this may be due to chromatin condensation because of the proliferation activity of cells. On the other hand, chromatin compaction may precede apoptosis in some cells [72]. Thus, the surface of the obtained PTFE/Al_2_O_3_-NPs composite coating containing 0.1 wt% Al_2_O_3_-NPs did not interfere with adhesion, growth, and normal development of primary cell cultures of mouse fibroblasts for 3 days.

## 4. Conclusions

A coating based on polytetrafluoroethylene and aluminum oxide nanoparticles was obtained. This coating had bacteriostatic properties and did not interfere with the adhesion and growth of eukaryotic cells. Such a coating may be useful for utilization as a filler in the surface of the coating and give them a long-term antibacterial effect. In particular, such coatings based on “liquid polytetrafluoroethylene” can find further application in the food industry.

## Figures and Tables

**Table 1:** Bacteriostatic properties aluminum oxide NPs reported in other studies.

№	Composition	Size, nm	Concentration	Type of Microorganism	Biological Effect	Ref.
1	Chitosan-coated Al_2_O_3_-NPs films	<50	0.05, 0.1 g/mL	*S. aureus*, *P. aeruginosa*, *S. epidermidis*	bacteriostatic	[56]
2	Chitosan/SiO_2_ nanocomposite with Al_2_O_3_	-	-	*S. aureus*, *P. aeruginosa*, *C. albicans*, *A. niger*	bacteriostatic	[57]
3	PLA/Al_2_O_3_	30	-	*P. aeruginosa* & *E. coli*	bacteriostatic	[58]
4	Al_2_O_3_/borosiloxane composite	45	0.001–0.1 wt%	*E. coli*	bacteriostatic	[59]
5	Al_2_O_3_–Ag composite nanoparticles	100–200	1–50 wt%	*E. coli & S. epidermidis*	bacteriostatic	[60]
6	Bulk Al_2_O_3_	100–200	MIC: 100 µg	*B. cereus*, *B. subtilis*, *K. pneumoniae*, *V. cholerae*	bacteriostatic	[61]
7	Bulk Al_2_O_3_	10–60	25–100 µg/mL	*E. coli*, *P. aeruginosa*, *S. aureus*	bacteriostatic	[62]
8	PANI–Al_2_O_3_ NPs composite	-	5, 10 mg/mL	*B. subtilis & E. coli*	bacteriostatic	[63]
9	Al_2_O_3_ coated by chitosan	80	0.025 mg/mL	*S. aureus*	bacteriostatic	[64]
10	Bulk Al_2_O_3_	<50	1–10 g/L	*B. cereus & P. stutzeri*	bacteriostatic	[65]

## Data Availability

The raw data supporting the conclusions of this article will be made available by the authors, without undue reservation.
